# Bempedoic Acid Treatment in Four Adult Patients with Glycogen Storage Disease Type 1a: A Retrospective, Exploratory Case Series

**DOI:** 10.3390/nu18193184

**Published:** 2026-09-26

**Authors:** Julya Hempel, Uta Meyer, Christian Menke, Charlotte Mindermann, Sabine Illsinger, Anibh Martin Das

**Affiliations:** Clinic for Paediatric Kidney, Liver and Metabolic Diseases and Neuropaediatrics, Hannover Medical School, Children’s Hospital, Carl-Neuberg-Str. 1, D-30625 Hannover, Germany; meyer.uta@mh-hannover.de (U.M.); menke.christian@mh-hannover.de (C.M.); mindermann.charlotte@mh-hannover.de (C.M.); illsinger.sabine@mh-hannover.de (S.I.); das.anibh@mh-hannover.de (A.M.D.)

**Keywords:** glycogen storage disease type 1, bempedoic acid, fasting time, lactate, lipids, uric acid

## Abstract

**Background:** Glycogen storage disease type 1a (GSD 1a) is an inborn error of metabolism due to glucose-6-phosphatase deficiency. Treatment consists of frequent, carbohydrate-rich meals, intake of glucose polymers like uncooked cornstarch, or drip feeding in small infants. Although dietary treatment is challenging, it has improved the long-term outcomes for patients with GSD 1a. Nevertheless, several complications, including dyslipidaemia, remain difficult to manage, and early atherosclerosis with risk for ischemic stroke is a potential long-term concern. We propose bempedoic acid (BA) as a potential treatment option for dyslipidaemia in adult patients with GSD 1a. BA is an approved lipid-lowering therapy and may have additional beneficial metabolic effects in patients with GSD 1a. We hypothesize that BA may reduce malonyl-CoA concentrations and thereby alleviate the inhibition of mitochondrial fatty acid oxidation. This could potentially contribute to improved metabolic stability in patients with GSD 1a. **Methods:** Four adult female patients with GSD 1a and dyslipidaemia received BA at a dose of 180 mg once daily for 2–5 years. In this retrospective case series, we explored potential additional effects of BA treatment beyond its established lipid-lowering effect. **Results:** Under BA treatment, patients reported prolonged fasting periods, and carbohydrate intake could be reduced in some patients. Plasma lactate concentrations decreased during treatment, while triglyceride concentrations decreased in two patients but increased in the others. Two patients experienced a slight, clinically irrelevant increase in serum uric acid concentrations, a known adverse effect of BA. No other adverse effects were observed. **Conclusions**: In this retrospective, uncontrolled, single-center case series, BA was associated not only with its expected lipid-lowering effect, but also with potential improvements in metabolic control and prolonged fasting tolerance in adults with GSD 1a. However, these preliminary observations require confirmation in larger, prospective, controlled studies. Future studies should include standardized metabolic assessments and direct measurements of key metabolites involved in the pathophysiology of GSD 1a. Preclinical studies using appropriate animal models may further contribute to elucidating the underlying mechanisms and potential metabolic effects of BA.

## 1. Introduction

Glycogen storage disease type 1 (GSD 1) was first described by von Gierke in 1929 based on autopsy reports of two children showing excessive amounts of glycogen in liver and kidneys [1]. It was not until 1952 that Cori and Cori described the deficiency of glucose-6-phosphatase as the underlying enzymatic defect in GSD 1 [2].

GSD 1 is a rare inborn error of metabolism with an incidence of approximately 1 in 100,000 live births. It is inherited in an autosomal-recessive manner.

Two subtypes of GSD 1 are known. GSD 1a is caused by variants of *G6PC* (encoding the enzyme glucose-6-phosphatase catalytic subunit), while variants of *SLC37A4* (encoding glucose-6-phosphate transporter) are responsible for GSD 1b [3].

Both the enzyme and the transporter are essential for the final step of gluconeogenesis and glycogenolysis, enabling the conversion of glucose-6-phosphate to glucose. In GSD 1a, the glucose-6-phosphatase in the endoplasmic reticulum (ER) is deficient, while in GSD 1b, the import of glucose-6-phosphate into the ER is impaired [1,3]. The reduced flux of glucose 6-phosphate results in the accumulation of glucose-6-phosphate in the cytosol. Glucose-6-phosphatase and the glucose-6-phosphate transporter are expressed mainly in liver, kidney, and intestine. Therefore, these organs are primarily affected by GSD 1.

Clinical hallmarks of GSD 1 are hepato- and nephromegaly, growth retardation, and symptoms of hypoglycaemia, which may result in coma and/or seizures [4]. In GSD 1b, neutropenia and neutrophil dysfunction as well as inflammatory bowel diseases are common [4].

Biochemically, hypoketotic hypoglycaemia, hyperuricaemia, elevated lactate and alanine levels, and hyperlipidaemia are observed [4]. The latter in particular is associated with the potential long-term concern of early atherosclerosis with risk for ischemic stroke [5].

Standard dietary treatment consists of frequent, carbohydrate-rich feeding and raw cornstarch (or other glucose polymers), especially at night [4]. Restriction of galactose, fructose, sucrose, and lactose is controversially discussed [4].

A unique biochemical feature of GSD 1 is the accumulation of malonyl-CoA from glucose-6-phosphate. Malonyl-CoA acts as a regulator of fatty acid synthesis and oxidation. It inhibits the enzyme activity of carnitine-palmitoyl transferase 1 (CPT1). Hence, long-chain fatty acids (LC-FAs) cannot cross the inner mitochondrial membrane, which is impermeable to LC-FAs [6,7]. As a result, ß-oxidation is hampered, leading to a lack of energy generation, especially in skeletal and cardiac muscle, while ketogenesis in the liver is compromised (Figure 1).

In an attempt to bypass the defective transport of LC-FAs into the mitochondrial matrix, we previously suggested the use of medium-chain fatty acids (MCTs), which can enter the mitochondrial matrix without using the carnitine-acylcarnitine shuttle (CAC) [6] (Figure 2). This dietary modulation had a beneficial impact on clinical and biochemical parameters. However, this presumably cannot fully compensate for the lack of LC-FAs in the mitochondrial matrix of GSD 1a patients.

Rossi et al. investigated the mitochondrial function in peripheral blood mononuclear cells (PBMCs) of GSD 1a patients and observed increased CPT1 expression and upregulation of various other mitochondrial enzymes [7].

These findings led to the assumption that the enzymatic capacity of fatty acid oxidation is maintained or even increased in GSD 1a patients. However, the influx of fatty acids into the mitochondrial matrix is inhibited by cytosolic malonyl CoA accumulation [8].

Recently, we proposed repurposing bempedoic acid for the treatment of patients with GSD 1 [9] (Figure 2).

Bempedoic acid was approved for the treatment of hypercholesterolaemia and mixed dyslipidaemia (in metabolic disorders) in Germany by the regulatory bodies (GBA, ‘Gemeinsamer Bundesausschuss’, ‘Federal Joint Committee’) in 2021. Therefore, its use in GSD 1 patients is in label. It is a prodrug, and when activated to bempedoyl-CoA by very-long-chain-acyl-CoA synthase (ACSVL 1), which can be found in liver tissue, it inhibits the ATP-citrate lyase (ACLY). ACLY cleaves cytosolic citrate generated by the citric acid cycle in the mitochondrium to acetyl-CoA. When ACLY is blocked, the de novo synthesis of cholesterol from acetyl-CoA and triglycerides from malonyl-CoA is inhibited [10].

When malonyl-CoA production is reduced, the block of LC-FA influx into the mitochondrial matrix is released, and LC-FA can undergo ß-oxidation in the hepatic mitochondria to produce ketone bodies and ATP. Furthermore, the synthesis of cholesterol and triglycerides is reduced [10] (Figure 2).

Bempedoic acid is well tolerated when used to treat dyslipidaemias [10]. The most common side effect is the increase in serum uric acid levels due to inhibition of the renal organic acid transporter 2 (OAT2) [11].

We hypothesized that bempedoic acid may improve metabolic control in GSD 1a, especially the maintenance of normal glucose levels and the capacity to produce ketone bodies during hypoglycemia in patients with GSD 1a, while lipid concentrations are reduced.

As all patients with GSD 1a described in this case series suffered from dyslipidaemia, we opted—after informed consent by the patients (shared decision)—to treat them with bempedoic acid as a therapy approved for this condition.

All patients gave ‘broad consent’, which is routinely discussed at admission with all patients treated in our hospital, which means that all data of routine analyses collected during their treatment can be used anonymously for research purposes.

## 2. Materials and Methods

### 2.1. Study Design and Study Population

At our center, we currently treat four adult patients with genetically confirmed GSD 1a, all of whom are female and aged 20–47 years. All four patients had dyslipidaemia associated with their underlying condition. After discussing the efficacy and potential risks of all approved pharmacological treatment options, all patients decided to initiate bempedoic acid therapy for the treatment of dyslipidaemia. The duration of treatment and follow-up under bempedoic acid therapy ranged from 2 to 5 years. One 18-year-old male patient with GSD type 1a declined bempedoic acid therapy because he was planning to relocate to another area to begin vocational training.

Patient #3 unexpectedly became pregnant four months after initiation of bempedoic acid therapy, despite being told that conception should strictly be avoided under bempedoic acid treatment, and discontinued treatment as recommended. She gave birth to healthy twins. Bempedoic acid therapy was resumed shortly after delivery, as the patient chose not to breastfeed. Patient #4 temporarily discontinued bempedoic acid treatment for three weeks after moving from her parents’ home to her own apartment, as she did not obtain a new prescription in time.

### 2.2. Treatment with Bempedoic Acid

We started treatment with bempedoic acid at a dose of 180 mg per day. Initially, the diet was not changed but could be adapted with reduction of carbohydrate intake under treatment.

### 2.3. Clinical and Biochemical Data

Clinical and biochemical data were retrospectively compiled from the medical records, prior to therapy (baseline), and under bempedoic acid treatment. Patients visited our outpatient clinics every 6–12 months.

Key metabolic parameters used to routinely assess metabolic control included serum glucose, plasma lactate, alanine, 3-hydroxybutyrate, and acetoacetate, as well as serum uric acid, triglyceride, and LDL-cholesterol concentrations. Due to the large catchment area of our center and the considerable distances travelled by patients to attend outpatient visits, blood samples were collected under routine, non-standardized conditions rather than at standardized times or in a standardized metabolic state. We compared the mean values from the three most recent consultations before bempedoic acid therapy with the mean values under treatment with bempedoic acid therapy. For patient #2, who transferred to our center at the beginning of bempedoic acid therapy, only one pretreatment value was available for comparison. Under bempedoic acid therapy, laboratory values were assessed approximately every 4 to 12 months with a duration of follow-up from 2 to 5 years.

Fasting duration was regarded as an important clinical outcome. All patients used continuous glucose monitoring (CGM). In accordance with the guidelines of the American College of Medical Genetics and Genomics, patients were advised to maintain blood glucose concentrations above 3.9 mmol/L (70 mg/dL) during the night and above 3.3 mmol/L (60 mg/dL) during the day [4].

A glucose concentration below 3.3 mmol/L (60 mg/dL) during the day and below 3.9 mmol/L (70 mg/dL) during the night indicated the end of the fasting period. To prevent hypoglycaemia, patients consumed a meal or raw cornstarch or other glucose polymers. During the day, patients continuously monitored their blood glucose levels using CGM and received an alarm from the device if their glucose concentration fell below the predefined threshold. At night, all patients consumed glucose polymers before bedtime and were woken either by an alarm clock after a predefined fasting period or by an alarm from the CGM device. Daytime and nighttime fasting durations were assessed by patient report at each outpatient visit or after review by our metabolic dietician, who assessed the respective CGMS-recordings between visits in our outpatient clinic.

During the regular clinical follow-ups, renal function, liver enzymes, and blood count were controlled and patients were asked for clinical side effects (e.g., gout, muscle symptoms), carbohydrate intake, intake of medium chain fatty acids (MCTs), clinical signs of hypoglycaemias, improvement of quality of life, and stamina; however, no validated instruments or objective testings were used.

No additional specific interventions or procedures were performed for the purpose of this (retrospective) analysis. All clinical data reported in this manuscript were obtained from routine follow-up and are covered by the broad consent obtained at our institution.

## 3. Results

### 3.1. Clinical Outcome

The general characteristics of the four individuals with GSD 1a are summarized in Table 1; a summary of treatment results is presented in Table 2.

Bempedoic acid was well tolerated; the patients reported no side effects (e.g., gout, muscle symptoms).

In all four patients, patient-reported prolongation of fasting time, maximum +2 h during the day and maximum +2 h during nighttime, were reported under bempedoic acid treatment. Patient #1 could reduce the intake of carbohydrates at night. Towards the end of the observational period, she could reduce the amount of glucose polymers during nighttime to 6 g/h compared to 10 g/h prior to bempedoic acid treatment.

No significant hypoglycaemic episodes were observed based on continuous home glucose monitoring regularly assessed by our metabolic dietician, and no apparent clinical symptoms of hypoglycaemia were reported by the patients under bempedoic acid treatment. All patients reported a subjective improvement in their quality of life; however, this was not assessed using a validated objective instrument. Patients also reported increased stamina and improved exercise tolerance during bempedoic acid treatment, although these subjective observations were not confirmed by objective standardized exercise testing.

### 3.2. Biochemical Parameters

To evaluate the biochemical outcomes under bempedoic acid treatment, we compared lactate levels before and under treatment. Blood was drawn randomly during visits to our outpatient clinics; a standardized blood sampling was not possible, as we have a large catchment area, and patients need to travel long distances. Additionally, all patients do work and/or have a family with small children, which makes timing of appointments difficult.

As shown in Table 2, lactate levels dropped in three patients after treatment was initiated (average reduction 52%, most impressively in patient #3 with a 75% reduction).

The alanine levels rose in all patients (Table 2).

As mentioned above, uric acid levels in serum may rise under bempedoic acid treatment due to inhibition of the renal organic acid transporter 2 (OAT2) [10]. In our cohort, three patients showed elevation of uric acid levels (maximum +40%) under bempedoic acid treatment (Table 2) without any clinical symptoms reported.

We compared the levels of LDL-cholesterol (LDL-C) and triglycerides (TGL) before and under treatment with bempedoic acid. Changes in triglyceride levels were very variable. In patients #1 and #4, triglyceride levels rose (+65 and +44%, respectively), whereas in patients #2 and #3, triglycerides levels dropped (−41% and −2%, respectively). With the exception of patient #4, LDL levels decreased during therapy.

Additionally, patient #4 interrupted bempedoic acid treatment for three weeks due to running out of medication after she left her parent’s home to start her academic studies in another city. During this time, she had several hypoglycaemias (clinically and documented by CGMS, minimum 2.2 mmol/L), her fasting time was shortened and she felt weak, and she was unable to exercise at all; while under bempedoic acid, she was able to exercise for 2 h daily. After three weeks, she restarted treatment. Her blood sugar levels stabilized again as assessed by CGM, she felt no longer weak, was able to exercise and had again a fasting time of 4 h during the day and about 5–6 h in the night.

Patient #3 interrupted bempedoic acid medication after becoming pregnant and immediately noticed a shorter fasting time. Shortly after she gave birth, she restarted treatment with bempedoic acid and could restore her fasting times.

## 4. Discussion

Despite succeeding in securing long-term survival in GSD 1a patients, dietary therapy is challenging and does not reliably prevent short-term hypoglycaemia and long-term complications like dyslipidaemia or overfeeding, possibly resulting in metabolic syndrome, growth failure, hepatic adenoma/carcinoma, and end-stage kidney disease in all patients.

Based on biochemical considerations, we have recently proposed a novel treatment-option for GSD 1a by repurposing bempedoic acid [9]. If shown to be effective, this could represent a promising treatment option for dyslipidaemia in patients with GSD 1a, with potential benefits beyond lipid control to improve metabolic stability and maintain euglycaemia.

In the present manuscript, we describe our first real-world clinical experience with BA in the treatment of dyslipidaemia in a cohort of four adult female patients with GSD 1a.

Bempedoic acid is an approved medication in the EU for the treatment of hypercholesterolaemia and mixed dyslipidaemia in adults. We compared the levels of LDL-cholesterol and triglycerides before and under treatment with bempedoic acid. Three patients showed LDL lowering, from minimum −3% to a maximum of −56% under treatment. Only one patient showed higher LDL-cholesterol (+33%) under bempedoic acid treatment, and the reason for this is not clear. After the initial rise of LDL-cholesterol, it dropped from visit to visit, and finally LDL-cholesterol dropped under the target value. The reason for this slow response is not clear.

Changes in triglyceride levels were heterogeneous. In patients #1 and #4, triglyceride levels rose (+65% and +44%, respectively), whereas in patients #2 and #3, triglyceride levels dropped (−41% and −2%, respectively). Blood samples were taken randomly during daytime without documentation of fasting or non-fasting state. Fasting and non-fasting/postprandial triglyceride levels differ significantly [12]. This is especially important for patients with GSD 1. In G6pc-/--mice, de novo fatty acid synthesis (which can be blocked by bempedoic acid) contributes substantially to hepatic triglyceride accumulation under normoglycaemic conditions, while fasting-induced hypoglycaemia results in higher rates of lipolysis in these animals, leading to enhanced triglyceride synthesis and reduction in very-low-density lipoprotein catabolism with elevated triglyceride synthesis rates [13]. Though patients were not overtly hypoglycaemic during their outpatient visits, lower glucose levels may have led to catabolism with increased rates of lipolysis. Interestingly, both patients with elevation of triglyceride levels under bempedoic acid therapy take MCTs, which could contribute to increased triglyceride levels. There were a number of confounding factors under bempedoic acid treatment like changes in diet and no standardized blood sampling relative to meals; thus, the metabolic effects under bempedoic acid treatment cannot clearly be ascribed to the medication. For the comparability especially of the lipid levels, but lactate and ketone bodies as well, standardized testing (non-fasting, fasting, or postprandial) is needed.

Under treatment, no clinical side effects were reported. During regular follow-up visits, no change in renal function, liver enzymes, or blood count were found. The rise in serum uric acid levels is a known biochemical side effect of bempedoic acid treatment. This is based on the inhibition of the renal organic anion transporter 2 (OAT2) by bempedoic acid [10]. On the other hand, hyperuricaemia is a biochemical finding in metabolically unstable GSD 1a patients. In our patients, three patients showed elevations of uric acid levels under treatment with bempedoic acid (Table 2), which were not clinically relevant.

Thus, the first therapeutic goal—controlling dyslipidaemia in patients with GSD 1a—appears to have been at least partially achieved, with no clinically relevant adverse effects observed during follow-up.

Based on increased hepatic fatty acid oxidation, which can theoretically be expected if our hypothesis holds true, bempedoic acid may lead to increased ketogenesis, which we observed in three patients, though fasting state was not standardized. Ketone bodies should ideally be determined after overnight fasting in a standardized fashion, which was not feasible in the outpatient setting.

Improvement of biochemical markers could only be partly observed in our case series. Blood samples were taken randomly in the outpatient setting. No standardized fasting tests were performed. Standardized measurements—ideally following a prolonged fast or during hypoglycemia—are important, particularly for certain parameters such as lactate, triglycerides, and ketone bodies.

We hypothesize decreased malonyl-CoA levels in hepatocytes under bempedoic acid treatment; however, this was not directly studied in our patient cohort. Tissue biopsies are ethically difficult to perform in humans. Animal models of GSD 1a may be useful to address this issue.

The most notable observation in this case series was that all four patients reported prolonged fasting periods during both night- and day-time. Although an extension of nocturnal fasting by a maximum of two hours may appear modest, it may allow for a longer period of uninterrupted sleep and thereby represent a meaningful improvement in quality of life. However, this was only a subjective observation of patients. Other subjective observations reported by the patients during bempedoic acid treatment—including improvements in overall quality of life, stamina, and exercise tolerance, as well as reduced carbohydrate intake—were not objectively assessed using validated instruments or standardized tests in this retrospective case series. Nevertheless, these observations are clinically relevant, and warrant further investigation in prospective studies using objective and validated outcome measures.

Our retrospective, exploratory single-center case series has several limitations. First, it was a retrospective study and the sample size was small. Our cohort of adult patients with GSD 1 consists solely of female patients; however, we think that it can be translated to male patients. No standardized fasting tests were performed, samples were taken randomly in the outpatient setting, and data such as fasting time, quality of life, stamina, and exercise tolerance were largely patient-reported. However, these observations are noteworthy. The use of bempedoic acid, an approved lipid-lowering medication for the treatment of dyslipidaemia, may offer the additional potential benefit of improving metabolic control in patients with GSD 1a. This could be particularly relevant given the current lack of causal therapies for patients with GSD 1a and a considerable unmet need.

In recent years, gene therapy and genome editing have been suggested as therapeutic options in GSD 1a [14]. Both preclinical and clinical studies were performed, and some were successful in terms of (partial) improvement of clinical symptoms and correction of biochemical parameters, while some therapeutic measures only resulted in temporary correction. In GSD 1b, gene therapy only addressed liver and kidney symptoms, but had no effect on haematological symptoms like neutropenia [14]. Safety issues with these therapeutic options have been reported [14].

## 5. Conclusions

In conclusion, bempedoic acid may represent a promising treatment option for patients with GSD 1a and dyslipidaemia, with the potential to improve lipid control and, in the best-case scenario, metabolic control and prolong fasting tolerance. Such an effect could have a meaningful positive impact on patient quality of life. However, prospective, long-term, multicenter studies are needed to confirm these findings and to further elucidate the full therapeutic potential and clinical implications of bempedoic acid treatment in patients with GSD 1a. Our retrospective case series in a single center gives initial promising data. Regarding the biochemical mechanism of bempedoic acid treatment in GSD 1a, direct measurement of malonyl-CoA in clinically relevant tissue like liver is essential to prove that our therapeutic hypothesis is correct.

## Figures and Tables

**Figure 1:**
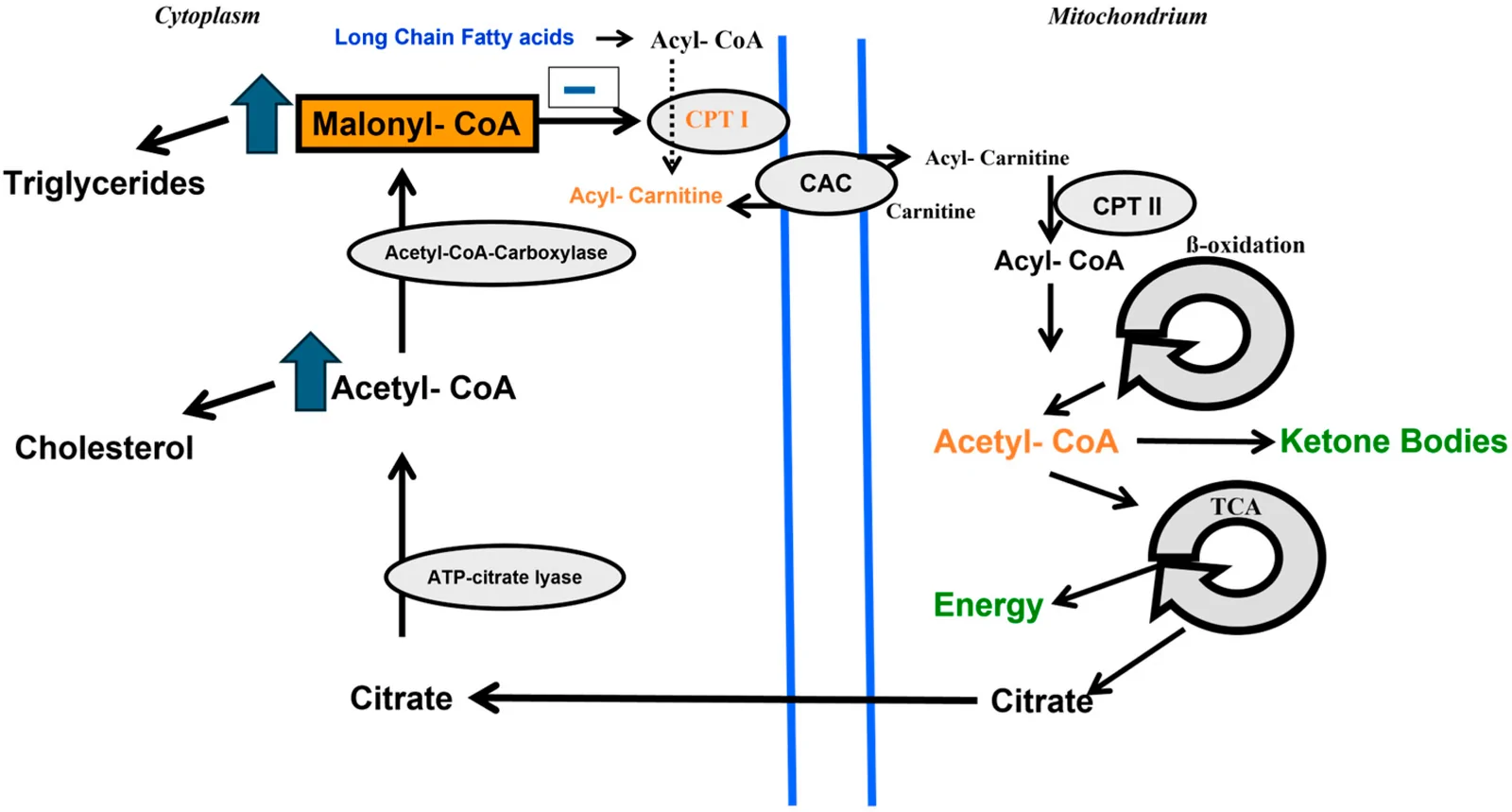
GSD 1 and fatty acid oxidation: In GSD 1, cytosolic acetyl-CoA is elevated, resulting in an increased synthesis of cholesterin and triglycerides. Increased cytosolic acetyl-CoA leads to elevated levels of malonyl-CoA. Thus, CPT1 is inhibited and influx of long chain fatty acids into the mitochondrial matrix is inhibited. CAC: carnitine-acylcarnitine shuttle, TCA: tricarboxylic acid cycle [3].

**Figure 2:**
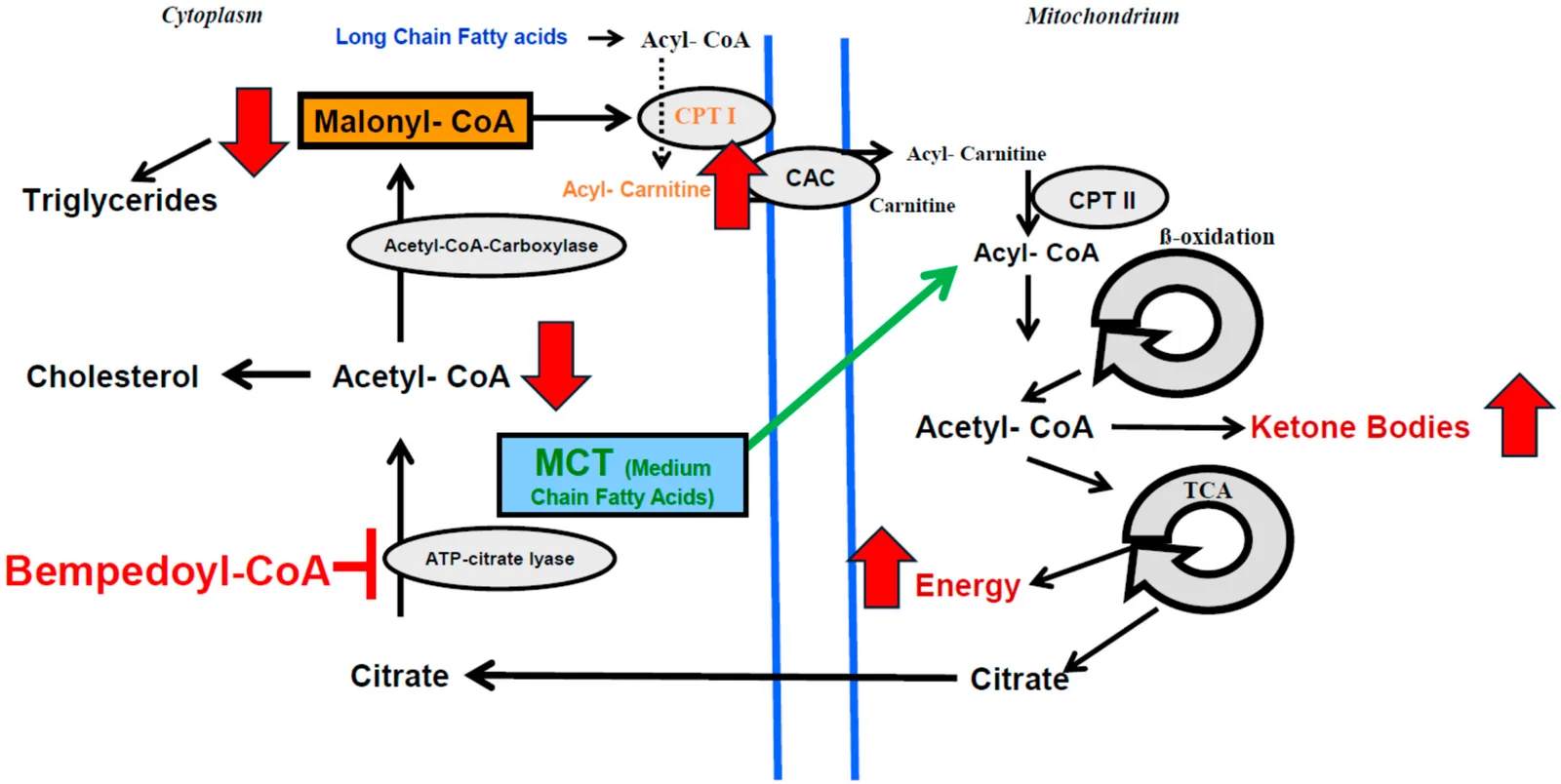
Therapeutic options in GSD 1: (1) MCTs (medium chain triglycerides) can enter the mitochondrial matrix without using the carnitine-acylcarnitine shuttle (shown in green). (2) Bempedoic acid is first activated to bempedoyl-CoA, which inhibits ATP-citrate lyase. Thus, cytosolic acetyl-CoA and malonyl-CoA concentrations presumably drop, leading to a release of CPT1 inhibition. Thus, the block of acyl-CoA transport into the mitochondrial matrix is released (shown in red).

**Table 1:** Patient characteristics.

Patient	1	2	3	4
**Sex**	F	F	F	F
**Age at start BA**	47 years	41 years	29 years	20 years
**Dose of BA**	180 mg	180 mg	180 mg	180 mg
**Other medications**	Ramipril L-thyroxine	Ramipril	Allopurinol, Enalapril	Enalapril
**Duration of follow-up on BA treatment**	5 years	2 years	3 years	4 years
**Relevant GSD 1a complication**	hepatomegaly	hepatomegaly	hyperuricaemia, hepatomegaly, steatosis hepatis	hepatomegaly
**BA: bempedoic acid**				

**Table 2:** Biochemical parameters before (−) and under treatment with bempedoic acid +(BA).

Patient	1		2		3		4	
	−	+BA	−	+BA	−	+BA	−	+BA
**Lactate mmol/L** **(mean)**	3.87	3.0 (−23%)	2.4 ^1^	1.2 (−50%)	11.6	2.9 (−75%)	2.53	3 (+20%)
**Uric acid µmol/L** **(mean)**	419	457 (+9%)	370 ^1^	448 (+20%)	537	495 (−8%)	298	421 (+40%)
**3-hydroxy-** **Butyrate µmol/L (mean)**	54.5	172 (+215%)	20.2 ^1^	51 (+150%)	n.d.	68	48	121 (+150%)
**Acetoacetate µmol/L (mean)**	16	87 (+400%)	10.5 ^1^	40.6 (+300%)	n.d.	44	48	56 (+17%)
**LDL-cholesterol** **mmol/L (mean)**	3	2.6 (−13%)	4.5 ^1^	1.9 (−56%)	3.8	3.7 (−3%)	2.5	3.3 (+33%)
**Triglycerides mmol/L (mean)**	3.2	5.3 (+65%)	4.4 ^1^	2.6 (−41%)	5	4.9 (−2%)	3.6	5.2 (+44%)
**Alanine µmol/L (mean)**	493	569 (+15%)	632 ^1^	882 (+40%)	490	573 (+17%)	477	630 (+30%)
**Fasting time Day** **in hours**	3	4 (+1)	3	5 (+2)	3	4 (+1)	2	4 (+2)
**Fasting time Night in hours**	6–8	8 (+2)	5	6 (+1)	4	5 (+1)	4	6 (+2)
**Clinical side** **effects**		No		No		No		No
**Nutritional assessment:** **carbohydrates g/h** **MCTs (g/d)**	10 10	6 10	12–140	90	120	n.d.0	n.d. 10–20	n.d. 10–20
**BMI kg/m^2^**	33	32	30	30	28	28	26	26

mean = Values represent the mean of three laboratory measurements, ^1^ only one pretreatment value, BA: bempedoic acid, MCTs: medium-chain fatty acids, LDL-cholesterol: low-density lipoprotein.

## Data Availability

The raw data supporting the conclusions of this article will be made available by the authors on request due to privacy.

## Abbreviations

The following abbreviations are used in this manuscript:

BA	bempedoic acid
CPT1	carnitine-palmitoyl transferase 1
GSD1	glycogen storage disease type 1
LDL-C	LDL-cholesterol
LC-FA	long-chain fatty acid
MCT	medium-chain fatty acid
OAT2	organic acid transporter
TGL	triglyceride
